# Immunisation program training needs in 9 countries in the African Region

**DOI:** 10.11604/pamj.2021.39.41.29492

**Published:** 2021-05-17

**Authors:** Balcha Girma Masresha, Carine Dochez, Ado Bwaka, Meseret Eshetu, Gilson Paluku, Richard Mihigo

**Affiliations:** 1 World Health Organisation, Regional Office for Africa, Brazzaville, Congo,; 2 Network for Education and Support in Immunization, University of Antwerp, Antwerp, Belgium,; 3 World Health Organisation, Inter-Country Support Team for West Africa, Ouagadougou, Burkina Faso,; 4 World Health Organisation, Inter-Country Support Team for East and Southern Africa, Harare, Zimbabwe,; 5 World Health Organisation, Inter-country Support Team for Central Africa, Libreville, Gabon

**Keywords:** Immunization, vaccination, training needs, capacity building, mid-level managers

## Abstract

**Introduction:**

regular in-service training of healthcare workers within the immunization program is critical to address the program needs created by the introduction of new vaccines and technologies, as well as the expanding scope of immunisation programmes beyond infant immunization and towards a life-course approach. National immunization programs conduct in-service training of health workers depending on program needs and particularly when new program elements are introduced.

**Methods:**

we conducted a survey of national and provincial level immunization program staff in 9 countries in the World Health Organization (WHO) African Region to determine the perceived needs and preferred training methods for capacity building in immunisation.

**Results:**

nearly all of the respondents (98.3%) stated that there are skill gaps at their respective levels in the immunization program which require training, with 88% indicating that mid-level program management (MLM) training was needed to train new program staff, while 78% indicated program performance gaps and 60% of the respondents stated that refresher training is needed. Program areas identified as top priorities for training included immunisation monitoring and data quality, sustainable immunization financing, adverse events monitoring and community mobilization. More than three quarters of the respondents (78%) think that online MLM training is adequate to address program gaps. Only four of the 9 immunization program managers indicated that they regularly monitor the number of MLM trained staff within their national program.

**Conclusion:**

there is a strong need for in-service training of immunization program officers in the countries surveyed, especially at the subnational levels. Program managers should conduct regular monitoring of the training status of staff, as well as conduct detailed training needs assessments in order to tailor the training approaches and topics. Online training provides an acceptable approach for capacity building of immunization program staff.

## Introduction

Countries in the African Region have introduced several new and underutilized antigens in their national immunization programs in the past two decades and have made significant progress in vaccination coverage [1]. However, following a period of steady increase from 2000 to 2009, the coverage levels for multiple antigens has not improved further in most of the countries in the region since then [2]. Immunisation program reviews conducted during the past decade indicate that gaps in the management of human, material and financial resources at district and health facility levels constitute one of the barriers to improving coverage and reaching every child [3,4]. Well-trained staff, which are sufficient in numbers, adequately deployed and motivated are crucial to effectively deliver high quality immunization services. The global vaccine action plan (GVAP) as well as the African regional strategic plan for immunisation (RSPI) both outline capacity building as a crucial pillar for the attainment of the objectives [5,6]. World Health Organisation has developed a framework to guide the global immunisation agenda from 2021 to 2030 which also puts strengthening immunisation systems as one of the key programmatic focus areas [7]. Regular in-service training of healthcare workers within the immunization program is critical to address the program needs as national programs introduce new vaccines and technologies, as well as expand their scope beyond infant immunization and towards a life-course approach. In addition, the high staff turnover in national immunization programs requires regular in-service training of professionals just joining the workforce from health training institutions or from other programs. However, in many countries, according to the authors´ personal observations, new officers joining the immunization program at subnational levels are not systematically offered a complete onboarding training, except during refresher trainings, or during programmatic events like the introduction of new vaccines.

Capacity building tailored for service providers at the operational level helps to improve service delivery, while the training of mid-level program managers will be key to improve managerial skills required to optimally utilize available resources and implement strategies. Accordingly, in the past two decades World Health Organisation had introduced the Immunization in Practice (IIP) set of training modules tailored for the service providers, and the mid-level management (MLM) course for immunization managers [8,9]. As training methods evolve over time, delivering the training using suitable techniques for each specific audience is critical in achieving good learning outcomes [10]. Between 2017 and 2019, the World Health organisation African Regional MLM course was revised to make it more relevant to program needs, and it was also modified to serve as an online self-paced training tool [11,12]. The results from the last 18 months following the launch of the online course indicate a wide acceptability and high demand for the online MLM training across the Region and beyond [13]. Many countries include in-service training of immunization officers into their annual program plans, but this may not always be guided by formal training needs assessments. This manuscript discusses a needs assessment conducted to understand the current demand and preferred training methods for capacity building in immunisation.

## Methods

We identified 9 countries in the WHO African Region to take part in the quantitative survey. The selection of the countries was purposive, to ensure representation from self-financing countries alongside countries which are eligible for funding support from The Global Alliance for Vaccines and Immunizations (GAVI), the vaccine Alliance; to include countries of varying population sizes, and from the various geographic subregions. The countries selected were Chad, Cote d´Ivoire, Democratic Republic of Congo (DR Congo), Gabon, Liberia, Malawi, Namibia, South Sudan and Togo. We worked with the immunization experts in each of the countries and compiled the list of national and provincial level immunization program staff, who work for the ministry of health and the key partner agencies. We sent structured questionnaires through an online polling platform and collected the responses from 12 - 22 October 2020. In addition, each of the national immunisation program managers received a second set of questionnaires to investigate their specific roles and the immunization program priorities vis-a-vis capacity building. The responses were tabulated and analysed.

## Results

**Figure 1:**
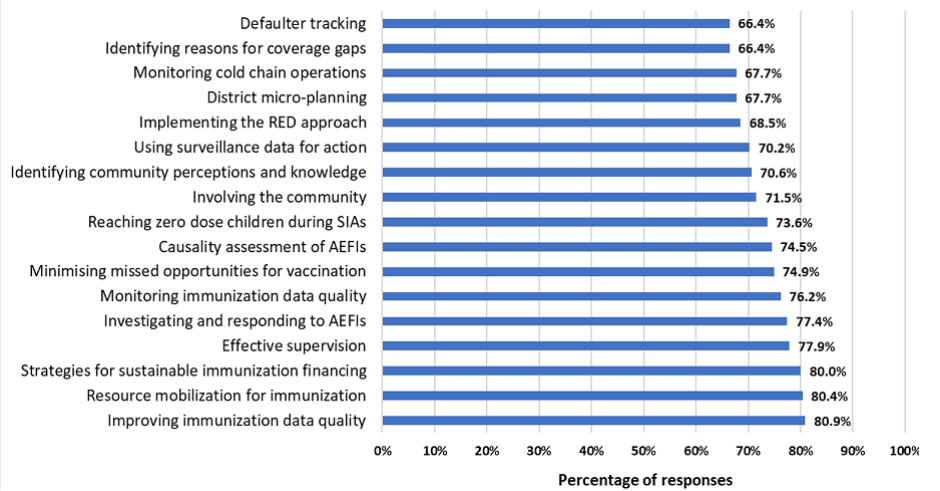
immunization program areas identified for training

**Training needs in immunization- the perception of immunization program officers in 9 countries:** a total of 241 immunization program staff from the 9 countries responded to the questionnaires, 88 of whom were from the national level (37%) while 95 were from the provincial (39.9%) and 55 were from the district levels (23.1%), while 3 participants did not indicate their level of work. Most of the respondents were public health experts (32.5%), disease surveillance officers (28.8%), or program data managers (8.8%). The length of service of the respondents in immunization programs ranged from less than 2 years (9.1%) to more than 10 years (47.3%) (Table 1). Nearly all of the participants (98.3%) stated that there are skill gaps at their respective levels in the immunization program which require training. Ninety one percent responded that their understanding of the training gaps was based on field observation or on supervisory findings while 68% also mentioned knowledge of program gaps, while only 28% stated it was based on some form of needs assessment. With regards to the reasons why MLM training was needed, 88% mentioned the need to train new program staff, while 78% indicated program performance gaps and 60% of the respondents stated that refresher training is needed since the last training was done too long ago (Table 2). The majority (58%) of the respondents had taken MLM courses in the past, while 132 (55.2%) had taken some online training courses before. Nearly two thirds of respondents (64%) were familiar with the WHO African regional online MLM training package. The majority (78%) of the respondents across all levels think that online MLM training is appropriate enough to address the program needs in their country, while 14% disagreed. Out of a total of 235 respondents in the 9 countries who indicated the areas identified for training, immunisation monitoring/ data quality, sustainable immunization financing, adverse events monitoring and community mobilization were identified as the broad program areas where most of the need existed at the time of the study (Figure 1). On the other hand, program areas like planning for new vaccine introduction, conducting cold chain inventory, monitoring cold chain operations, budgeting for and costing immunization activities, implementing active surveillance, ensuring vaccine safety during supplementary immunization activities (SIAs), among others, were prioritized less (Table 3).

**Table 1:** demographic characteristics of respondents

		Responses	Percentage
**Country**	Chad	39	16.3%
	Cote D'Ivoire	13	5.4%
	DR Congo	60	25.0%
	Gabon	17	7.1%
	Liberia	22	9.2%
	Malawi	19	7.9%
	Namibia	12	5.0%
	S Sudan	44	18.3%
	Togo	14	5.8%
	**TOTAL**	**240**	**100.0%**
**Level of professional engagement**	National	88	37.0%
	Provincial	95	39.9%
	District	55	23.1%
	**TOTAL**	**238**	**100.0%**
**Professional responsibility**	Immunization program Manager	10	4.2%
	Public health expert	78	32.5%
	Logistician	10	4.2%
	Data manager	21	8.8%
	Communications	8	3.3%
	Surveillance	69	28.8%
	other	44	18.3%
	**TOTAL**	**240**	**100.0%**
**Years of service in the immunization program**	less than 2 years	22	9.1%
	2 - 5 years	54	22.4%
	6 - 10 years	51	21.2%
	more than 10 years	114	47.3%
	**TOTAL**	**241**	**100.0%**

**Table 2:** reasons provided to justify the need of MLM training in the 9 countries

Reason why MLM training may be needed	Responses	Percentage
Need to train new program staff/ high staff turnover	213	88.80%
Performance gaps to improve	187	77.90%
Too long since last training - refresher training needed	143	59.60%
Introduction of new technology	121	50.40%
New program priorities in the coming months and years	88	36.70%
New administrative units/ districts created	78	32.50%
Others	18	7.50%

**Table 3:** immunization program topics prioritized for MLM training by respondents

Program area	Area identified for training need	Percentage
**Planning**	district micro-planning	67.7%
	identifying reasons for coverage gaps	66.4%
	defaulter tracking	66.4%
	identifying hard-to-reach populations	58.3%
	immunization program operational planning	53.6%
	general problem solving	46.4%
	strategic planning	45.1%
	planning for introducing new vaccines	39.1%
**Communications**	involving the community	71.5%
	identifying community perceptions and knowledge gaps	70.6%
	conducting advocacy for immunisation	58.7%
	immunization program stakeholder analysis	58.7%
	identifying communications barriers	58.3%
	developing messaging for social mobilisation	44.3%
**Financing**	resource mobilization for immunization	80.4%
	developing strategies for sustainable financing of immunisation	80.0%
	budgeting immunization activities	51.1%
	calculating immunization program costs	42.1%
**Logistics**	monitoring cold chain operations	67.7%
	managing vaccine stocks	62.6%
	estimating and forecasting vaccine needs	57.0%
	estimating cold chain space needs	57.0%
	doing a cold chain inventory	43.0%
**Monitoring**	improving immunization data management and data quality	80.9%
	monitoring immunization data quality	76.2%
	establishing immunisation program monitoring systems	57.0%
	analysis and interpretation of immunization monitoring data	57.0%
	selecting monitoring indicators	40.0%
**Vaccine safety**	investigating and responding to AEFIs	77.4%
	causality assessment of AEFIs	74.5%
	ensuring vaccine and injection safety in daily vaccination activities	53.2%
**Supplemental immunization activities**	reaching zero dose children during SIAs	73.6%
	monitoring readiness of SIAs	58.7%
	SIAs planning	58.3%
	ensuring vaccine safety during SIAs	49.8%
**Surveillance**	using surveillance data for immunisation program action	70.2%
	improving VPD surveillance performance	64.7%
	mobilising funding for surveillance and laboratory	64.7%
	investigating outbreaks of vaccine preventable diseases	54.9%
	analysing and interpreting surveillance data	53.6%
	implementing active surveillance	51.1%
**Supervision**	conducting effective Supportive supervision	77.9%
	providing supervisory feedback	63.0%
	prioritizing for supervision	57.0%
**Other operational areas**	minimising missed opportunities for vaccination	74.9%
	implementing the RED approach	68.5%
	planning immunisation program reviews	56.2%
	establishing vaccination in the second year of life and beyond	54.5%

**Capacity building in immunisation-the national program perspective:** only four of the 9 immunization program managers indicated that they regularly monitor the number of MLM trained staff within their national program. The last MLM national and subnational training was conducted more than 4 years back in 4 of the 9 countries. Immunization in practice (IIP) training was conducted within the last 2 years in 5 countries, while one program manager stated that IIP training was done 5 years ago. Seven of the 9 program managers estimated that less than half of the national immunization program staff were trained in the mid-level managers´ (MLM) course. At province level, less than half of the program staff had taken the MLM course in 5 countries, while this was true at the district level in 7 countries. Two of the program managers responded that they did not know the proportion trained at province and district levels. The proportion of immunization program staff with access to internet connection and able to take online training was estimated to be less than 50% at province level in one country, while it was less than 50% at district level in 4 countries. In addition to traditional approaches of in-service training, 7 of the 9 program managers stated that they have at some time utilized mass messaging platforms (WhatsApp and/or Telegram apps) to provide training content on immunization to health workers. Six of these stated that they used these approaches for training around special program activities.

## Discussion

In the past two decades, there have been rapid developments in the uptake of new and underutilized vaccines, in expanding the scope of immunization programs beyond infancy and in the introduction of new tools and technologies for cold chain and immunisation monitoring. There are still multiple new vaccines in the pipeline for countries in the Region to consider in the coming years. Within this dynamic context, it is important to ensure that health workers are regularly trained and updated to be able to meet the changing demands. Lafond *et al*. have identified the regular review of programme and health worker performance as one of the six key drivers of routine immunization coverage [14]. In our study, nearly all of the respondents (98.3%) stated that there are skill gaps at their level in the immunization program which require training. In this study, the reported proportion of immunization program staff that have taken the MLM course was less than half at subnational levels in most of the countries. Immunisation program reviews in various countries have indicated skills gaps and training needs [3]. However, there are very few published immunization training needs assessments in the literature [15-17]. It may not be feasible to do comprehensive training needs assessments regularly. In such cases, program managers can use information from various sources (triangulating between emerging program needs, supervisory observations from the field, reports of recent program reviews, as well as data from formal and informal needs assessment exercises) to determine the focus for in-service training. In this study, some of the program areas identified as highly relevant for training include immunization data management and data quality, identification and management of adverse events following immunisation (AEFIs), conducting effective supervision, minimizing missed opportunities for immunisation. These priority areas are similar to the gaps documented in a training needs assessment in Nigeria [18]. On the other hand, program areas like the introduction of new vaccines, cold chain inventory and strategic planning received the lowest prioritization. This may be related to recent trainings in these countries addressing these program areas.

We found that only 4 of the 9 immunization program managers regularly monitor the number of MLM trained staff within their national program. Moreover, the last MLM national and subnational training was conducted more than 4 years back in 4 of the countries. These findings indicate the need for a more systematic approach to the capacity building of immunisation professionals. National program managers need to choose which of the various courses fit the profile of health workers where the skills gaps need to be addressed. The recent overhaul of the WHO Afro MLM training tools provides the flexibility to use the tools online as well in workshop-based trainings at national and subnational levels, and provides flexibility to select the modules most relevant for the program´s training needs [15].

In recent years, the expansion in internet connectivity in African countries and the development of online training tools have increased the access to and acceptability of online training courses. More than half of our study respondents have taken some online courses in the past, and four out of five think that online MLM training can be used to address the training needs in their country. However, it is noted that limitations to internet access, especially at the district level, may continue to pose challenges to wide-scale launch of online training courses. While the design of the MLM course covers most of the basic elements, the development of skills requires additional training approaches like simulation exercises and drills [19]. Other studies have also indicated the utility of supportive supervision for optimal immunisation program performance, including in areas like program data management [20,21]. The use of mass messaging platforms for the supervision and training of health workers has already proven to be a useful alternative to consider in low resource settings [22]. At least 7 of the 9 countries in this study have at some time utilized mass messaging platforms for distributing immunization training content to health workers. The utility and the experience of mass messaging platforms for sharing training content needs to be explored further and lessons documented.

The introduction of COVID-19 vaccines in early 2021 has come with its own demands for health worker training. The pandemic context, the introduction of new vaccine products in use, the different cold chain requirements, the specific population groups that often are not targeted in mass vaccination activities, the need to implement infection prevention and control measures, and the phased approach to implementation render the trainings needs different from previous ones [23]. However, at the same time, such occasions present programmatic focus, as well as opportunities and resources that can be used to build specific technical skills as well as transferable managerial skills among program staff. In the context of the COVID pandemic, many countries and partner agencies have scaled up online training sessions and webinars as a means of remote assistance to build national program capacity.

**Limitations:** Our study did not attempt to do an objective analysis of skills gaps. It was aimed at capturing the perceptions of immunisation professionals in the selected countries through a self-reported prioritization of needs. The findings have been presented in an aggregated manner, and attempts were not made to compare the results from the various countries, as there was a wide variation in the number of respondents across the selected countries.

## Conclusion

There is a strong need for in-service training of immunization program officers in the countries surveyed. However, detailed and country-specific training needs assessments should be done in order to tailor the training approaches and topics to serve the program needs. National immunization program managers should monitor the training levels of staff at national as well as subnational levels. Online training provides an acceptable approach to build the capacity of mid-level program managers.

### What is known about this topic

- National immunization programs continue to experience high staff turnover and an expansion of program scope, requiring regular training of health workers;
- Immunisation program reviews in various countries have indicated skills gaps and training needs;
- Online training courses have provided additional opportunities for capacity development.

### What this study adds

- Immunisation program staff at various levels perceive that major skill gaps which require the training of health workers persist;
- The proportion of immunization program staff at subnational levels that have taken the MLM training course was less than half in most of the countries;
- Online training and mass messaging platforms provide acceptable alternative ways of extending training content to program staff; country level training needs assessment should be done regularly in order to tailor the focus of training and to determine the best approaches to serve the program needs.
